# Unlocking cellular traffic jams: olive oil-mediated rescue of CNG mutant channels

**DOI:** 10.3389/fphar.2024.1408156

**Published:** 2024-07-25

**Authors:** Angeles Avalos-Hernandez, Karina Juarez-Navarro, Estela Ruiz-Baca, Ivan Meneses-Morales, Edith Espino-Saldaña, Ataulfo Martinez-Torres, Angelica Lopez-Rodriguez

**Affiliations:** ^1^ Facultad de Ciencias Químicas, Universidad Juárez del Estado de Durango, Durango, Mexico; ^2^ Laboratorio de Neurobiología Molecular y Celular, Instituto de Neurobiología, Universidad Nacional Autónoma de México. Campus Juriquilla, Juriquilla, Mexico

**Keywords:** ion channel, trafficking, electrophysiology, lipids, plasma membrane

## Abstract

One of the reasons to suggest olive oil consumption for a healthy life is its potential to induce robust lipidomic remodeling through membrane modification by dietary lipids. This remodeling might, in turn, modulate essential lipid-protein interactions while maintaining accurate transmembrane protein/domain orientation. Oleic acid, the primary compound in olive oil, has been suggested as a modulator of ion channel function. In this study, we explored whether this lipid could rescue the trafficking of mutated transmembrane proteins. In our initial approach, we supplemented the cell culture medium of HEK-293 cells expressing cyclic nucleotide channels tagged using green fluorescent protein (CNG-GFP) with olive oil or oleic acid. In addition to wild-type channels, we also expressed R272Q and R278W mutant channels, two non-functional intracellularly retained channels related to retinopathies. We used fluorescence microscopy and patch-clamp in the inside-out configuration to assess changes in the cell localization and function of the tested channels. Our results demonstrated that olive oil and oleic acid facilitated the transport of cyclic nucleotide-gated R272Q mutant channels towards the plasma membrane, rendering them electrophysiologically functional. Thus, our findings reveal a novel property of olive oil as a membrane protein traffic inductor.

## Introduction

About 98% of *Olea europaea*, a fruit commonly known as olive, is made up of a saponifiable-lipophilic fraction. The lipidic component is 55%–83% oleic acid (C18:1), a monounsaturated omega-9 fatty acid, and 3.5%–21% linoleic acid (C18:2), 7.5%–20% palmitic acid (C16:0), 0.5%–5% stearic acid (C18:0), and 0%–1.5% alpha-linolenic acid (C18:3) (Blekas et al., 2006; Farooqui, 2012; Lopez et al., 2014). Most of these lipidic compounds can be used as structural elements within the cell, particularly as free fatty acids, phospholipid precursors for membranes, and within lipid droplets (Brasaemle and Wolins, 2012; Sturley and Hussain, 2012). Lipid droplets are intracellular organelles with a dynamic structure that function in lipid storage, transportation, and intracellular signaling (Rohwedder et al., 2014; Nakajima et al., 2019; Eynaudi et al., 2021).

Extensive molecular and biophysical studies have revealed that some interactions between fatty acids and ion channels may regulate protein traffic, structure, and function. However, the effects seem to vary depending on the cell type, fatty acid length, saturation, and intrinsic characteristics of the protein. Nevertheless, membranes enriched with oleic acid are more flexible, demonstrating higher elasticity and increased curvature (Ibarguren et al., 2014; Randall et al., 2015; Kurniawan et al., 2017). The physiological response to oleic acid-induced membrane modification varies and may have physiological benefits in different contexts or pathologies. For example, the blocking or inhibition of ion channels, such as TRPV1 or TMEM16A/ANO1, has been associated with a decrease in pain and inflammation responses, among other effects (Morales-Lazaro et al., 2016; Leon-Aparicio et al., 2022). Conversely, interactions between oleic acid and TRPC3/6 channels in human Jurkat T cells induce an increase in calcium currents, which has diverse effects, including modulation of the immune system response and control of cell proliferation (Carrillo et al., 2012).

Many transmembrane proteins are tightly regulated by membrane interactions, and modification of membrane complexity can have physiological consequences (Weijers, 2012; Bolognesi et al., 2013; Vandebrouck and Ferreira, 2020). A better understanding of the molecular mechanisms by which membrane lipid composition regulates the location and function of transmembrane proteins is imperative to designing alternative treatments for diseases in which the structure of membrane proteins is compromised, such as Menkes disease, cystic fibrosis, hereditary long QT syndrome, or achromatopsia, among other pathologies (Juarez-Navarro et al., 2020).

To investigate the role of olive oil as a membrane protein traffic and function modulator, we supplemented HEK-293 cell medium with either complete olive oil or oleic acid in an attempt to rescue two mutant cyclic nucleotide-gated (CNG) channels linked to achromatopsia reported as intracellularly retained dysfunctional proteins (Faillace et al., 2004). CNG channels are formed by four subunits, each containing six transmembrane segments (S1-S6) and an intracellular carboxy-terminal region that is essential for binding cyclic nucleotides (Xue et al., 2022). In the present study, we assessed the effects of oleic acid on the CNG R272Q and R278W mutant channels, where the second and fourth, respectively, of the regularly-spaced positively-charged amino acids in the S4 transmembrane segment are mutated (Kohl et al., 1998; Wissinger et al., 2001; Faillace et al., 2004; Michalakis et al., 2018). In voltage-gated potassium (Kv) channels, S4 is an integral part of the voltage sensor domain. It interacts with phospholipids in a thermodynamically favorable way (Bond and Sansom, 2007; Hite et al., 2014) and moves among membrane lipids in synchrony with membrane potential changes to control channel opening (Horn, 2004; Wee et al., 2011; Marchesi et al., 2012). CNG channels are activated through ligand interaction, and voltage sensitivity remains controversial. Nonetheless, keeping the structural integrity of S4 is essential since mutations in these segments yield intracellularly retained non-functional channels (Faillace et al., 2004).

## Methodology

### Mutagenesis

Green fluorescent protein tagged CNGA1 (CNG-GFP) was obtained by cloning the bovine CNG coding sequence (Bcnga1, gene accession number NM_174278.2) into the pAcGFP1-N2 vector (Addgene). Mutations in CNG-GFP channels were engineered by site-directed mutagenesis using a QuikChange kit (Stratagene). All amino acid substitutions (and the remaining coding sequence) were confirmed by DNA sequencing.

### Cell transfection

The HEK-293 cells were cultured in Dulbecco’s Modified Eagle Medium (with 10% fetal bovine serum, penicillin/streptomycin, and antibiotic/antifungal) at 37°C under 5% CO_2_. To facilitate cell attachment for subsequent experiments, the cell culture dishes contained sterilized glass coverslips pre-treated with poly-L-lysine (Sigma-Aldrich) and then washed with Phosphate-buffered saline (PBS) buffer. For the low-confluence cell cultures, cells were transfected with Lipofectamine 3000 reagent (Invitrogen) and a 2:1 mix of the vector expressing CNG-GFP channels (either wild type or mutant) and the mCherry-Mem vector, which expresses a plasma membrane marker (Yost et al., 2007). For lipid supplementation, the cell culture medium was exchanged 4 h after transfection with fresh medium supplemented with olive oil (Sigma-Aldrich, O1514, highly refined, low acidity) or oleic acid (Sigma-Aldrich, O1257 dissolved in H2O). Treated and untreated cells were maintained in culture for 48–72 h for imaging and electrophysiology.

### Epifluorescence microscopy

Micrographs were captured with an epifluorescence microscope (Nikon ECLIPSE TS2) using a ×20 objective and appropriate spectral filters. Cell cultures were exposed to a LED light beam of 475 nm excitation. Emitted light at 505 nm was used to detect CNG-GFP. Light was filtered at 587 nm excitation/610 nm emission to detect m-Cherry and at 530 nm excitation/635 nm emission for Nile Red fluorophore. All fluorescence images were analyzed using Fiji, ImageJ software (https://imagej.net/software/fiji). Co-localization analysis was performed using ImageJ (http://rsb.info.nih.gov/ij) and the JaCoP plug-in (Bolte and Cordelieres, 2006); for pairwise comparison of protein location, we used Mander’s overlap coefficient (Manders et al., 1992).

### Electrophysiology

Glass coverslips with HEK-293 cells attached were removed from the cell culture medium using forceps and placed into an individual culture plate containing intracellular solution (140 mM NaCl, 5 mM KCl, 10 mM HEPES, pH: 7.4). Electrodes were made from borosilicate glass capillaries (Harvard Apparatus) using a vertical puller model ListMedical L/M-3P-A with diameters between 8 and 15 μm and with a resistance of 3–5 MΩ. Using a syringe coupled to a tip model MF28G-5 (World Precision Instruments), an electrode was filled with extracellular solution (140 mM NaCl, 5 mM KCl, 10 mM HEPES and 2 mM CaCl_2_, pH 7.4). CNG channels are known to be sensitive to changes in pH. By keeping the pH consistent across both solutions, we ensured stable conditions, reducing the likelihood of pH-induced variability in channel activity (Morrill and MacKinnon, 1999). Channel currents were measured under inside-out patch-clamp configuration with an Axopatch 200B amplifier (Axon Instruments) and pClamp 9.2 software (Axon Instruments). Analog signals were filtered between 5 and 10 kHz. Currents were acquired in response to a 30 ms voltage step at −60 mV or +60 mV (the latter achieved by a 120 mV increase) from a holding potential of 0 mV. Calcium currents were recorded before applying cGMP, after applying cGMP, and after washing out cGMP (data not shown).

Current recordings were plotted as current density using the next formula:

$$\frac{Isus}{Icap}$$

Where:

**Isus**, measured in picoamperes, is the subtracted current (current after cGMP addition minus current before cGMP application).

**Icap** is the capacitive current and represents the product of capacitance (C) by the rate of voltage change over time (C*dV/dT), where capacitance (C) shows capacitor size; C= charge (Q)/Voltage (V), measured in picofarads.

Charge (Q) is calculated as follows:

$Q=\int I\left(t\right)\text{dt}$ , and it is expressed in Coulombs (C).

### Statistical analysis

To compare the co-localization of CNG-GFP and mCherry-Mem proteins or the current density, data are expressed as mean ± SEM. A one-way ANOVA was performed to provide a comprehensive analysis of variability across tested conditions, followed by a Tukey’s *post hoc* test (differences with a *p* < 0.05 were considered statistically significant).

## Results

To investigate the biological roles of olive oil derivatives as active mediators in membrane trafficking events, we evaluated their effects on the homomeric bovine CNGA1 channel tagged with GFP. This channel is a well-characterized CNG channels, with a wealth of existing biophysical data, and it shares high homology with human CNGA3 (Faillace et al., 2004; Lopez-Rodriguez and Holmgren, 2012; Barret et al., 2022). The experiments were conducted on HEK-293 cells individually supplemented with either olive oil or oleic acid. Amino acid residues R272 and R278 in the bovine CNGA1 channel are equivalent to R296 and R302 in the bovine CNGA3 channel. Mutations at these positions produced dysfunctional channels that were intracellularly retained when expressed in tsA-201 cells (a HEK-293 cell line stably expressing an SV40 temperature-sensitive T antigen) (Faillace et al., 2004). As shown below, similar results were obtained in HEK-293 cells expressing bovine CNGA1 harboring either the R272 mutation or the R278 mutation.

We performed dose-response experiments investigating the effects of supplementation with olive oil or oleic acid on channel trafficking and channel function. The concentrations of these additives were carefully chosen based on their ability to affect channel localization. At high concentrations (>4% olive oil or >0.25% oleic acid), these additives were insoluble and toxic for the cells. At low concentrations (<0.5% olive oil or <0.05% oleic acid), the additives had no effect on channel localization.

**FIGURE 1:**
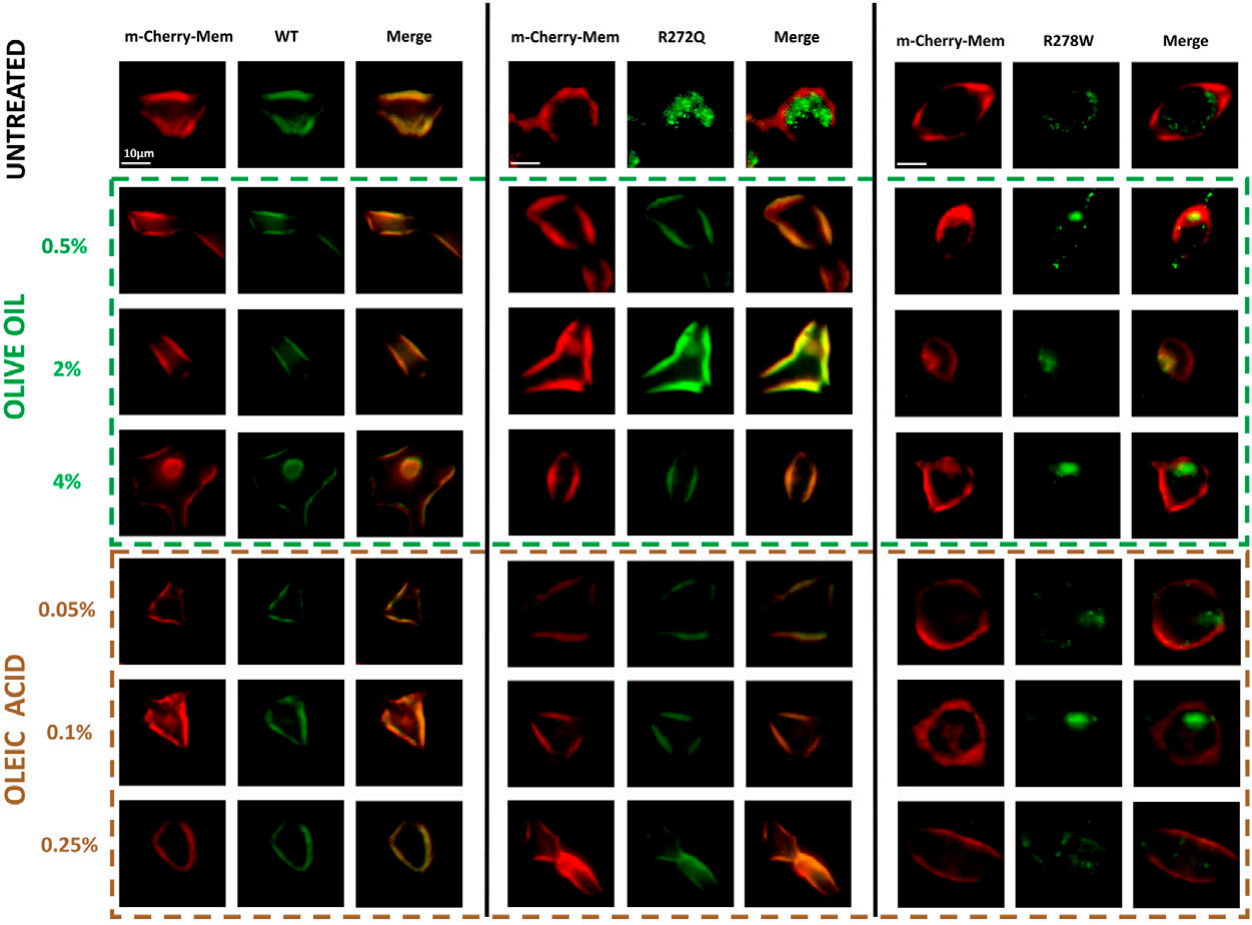
Effect of olive oil or oleic acid on the cellular localization of CNG channels. Micrographs of HEK-293 cells untreated or treated with different concentrations of olive oil or oleic acid, expressing distinct versions of CNG channels (CNG-GFP); a plasma membrane marker (m-Cherry); and the merge of both micrographs. n = 5.

**FIGURE 2:**
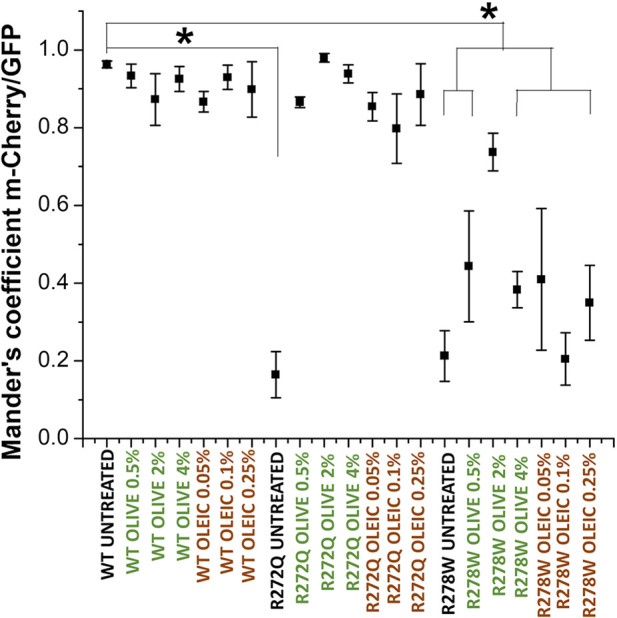
Analysis of the cellular location of CNG channels. Mander’s analysis shows co-occurrence of m-Cherry on GFP-tagged proteins [CNG wild-type and mutant channels (R272Q and R278W)]. Mander’s coefficients were determined for each cell (n = 5). Data are shown as mean ± SEM. Statistical analysis was performed in OriginPro 8. One-way ANOVAs and Tukey tests with a significance * indicate *p* < 0.05 compared to the WT control without treatment.

Firstly, we analyzed the cellular localization of CNG channels tagged with GFP in relation to the red-fluorescent plasma membrane marker (m-Cherry-Mem). We then evaluated electrophysiological responses to 10 mM cGMP for each condition. In Figure 1, we show images of the cells co-transfected with different versions of CNG-GFP and the m-Cherry-Mem vector, in the presence and absence of olive oil or oleic acid. Although the shuttling of the R272Q mutant CNG channel to the plasma membrane was noticeable, Mander’s co-localization coefficient was calculated to detect overlapping pixel colors as an indicator of protein co-occurrence within a complete single cell. Secondly, we evaluated in Figure 2, the overlap of m-Cherry with GFP pixels (M1) is plotted for treated and untreated CNG channels. This demonstrates that the degree of colocalization with the membrane marker was statistically different between cells expressing wild type (WT) channels or cells expressing R272Q channels treated with olive oil or oleic acid and untreated cells expressing R272Q channels. Similarly, the degree of colocalization with the membrane marker was statistically different between cells expressing wild type (WT) channels and cells expressing R278W mutant channels (no matter the treatment). Interestingly, the results also suggest a slight improvement in the intracellular trafficking of R278W channels as the concentration of olive oil increased. To investigate this further, we performed a statistical Pearson analysis and calculated Mander’s co-localization coefficient for the overlap of GFP with m-Cherry pixels (M2). The results reveal a significant difference between the co-localization of all analyzed R278W channels (and untreated R272Q channels) and the co-localization of untreated WT channels (Supplementary Figure 1).

**FIGURE 3:**
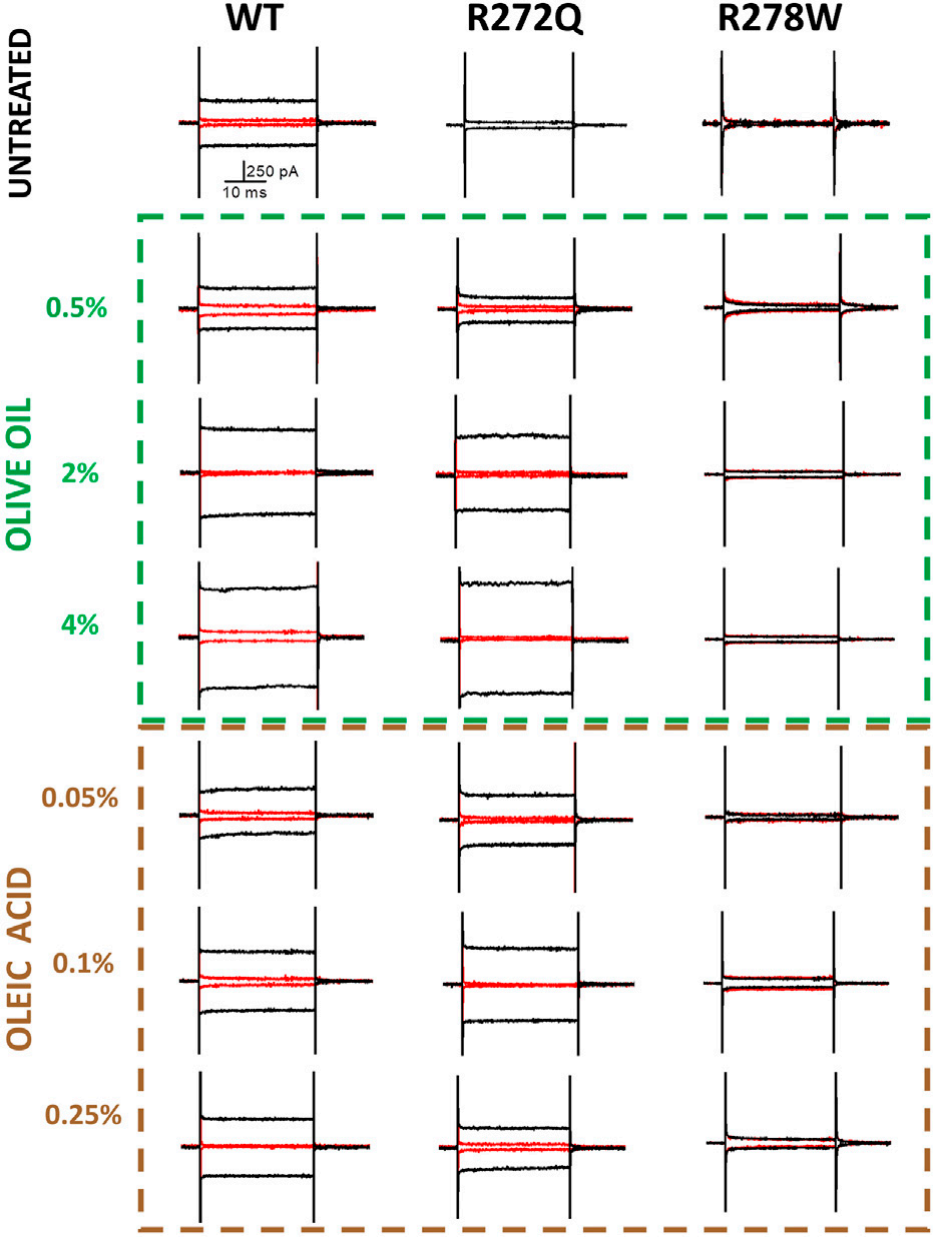
cGMP-dependent currents in the presence of olive oil or oleic acid. Membrane potential was held at 0 mV to give a pulse at −60 mV followed by a second pulse of +120 mV to reach +60 mV. In red, current before applying cGMP (10 mM); in black, current after ligand perfusion. Representative recordings. n = 4.

The experimental demonstration of electrophysiological activity in rescued channels can be used as evidence that a mutation that affects protein assembly and trafficking does not compromise domain function (Koeppen et al., 2008; Reuter et al., 2008; Duricka et al., 2012; Lopez-Rodriguez and Holmgren, 2012). Figure 3 shows representative traces of electrophysiological currents, with the black trace representing a cGMP- evoked current and the red traces representing currents in the absence of cGMP.

Consistent with the previous report by Faillace et al. (2004), the expression pattern of analyzed channels was characterized by only two types: functional channels located at the plasma membrane (co-localizing with m-Cherry-Mem fluorescence in Figure 2) and intracellularly retained and dysfunctional mutant channels.

Although our remarkable demonstration that olive oil and oleic acid can rescue membrane protein trafficking provides some evidence of the functionality of these channels, we also wanted to compare calcium current amplitudes between the rescued and WT channels.

**FIGURE 4:**
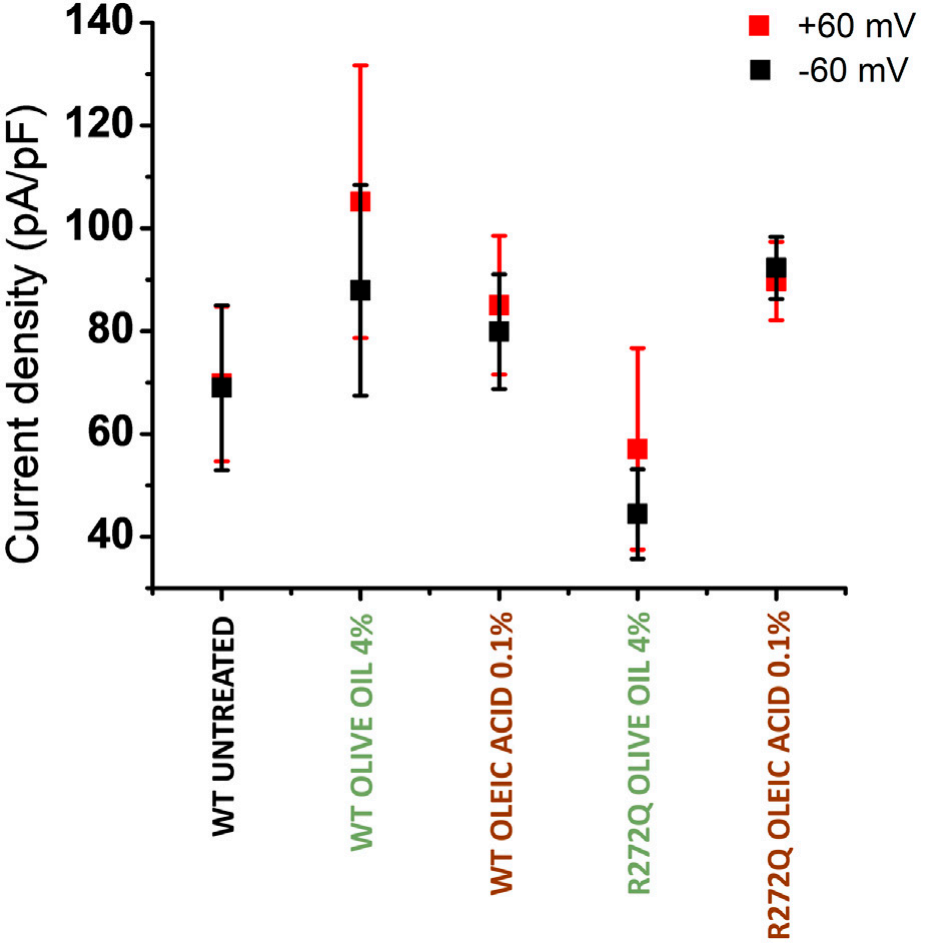
Current density of rescued CNG channels. Current density averages evoked at −60 mV (in black) and +60 mV (in red) ± SEM. n = 4.

As shown in Figure 3, the representative current traces appear to demonstrate some dependence on the concentration of additive. However, the calcium currents under each condition are likely to vary due to multiple factors, including the level of protein expression per cell or per patched area. To facilitate a better comparison of functionality across different CNG channel populations, we calculated the current density of rescued mutant channels. Figure 4 depicts the current density on cells expressing WT channels (untreated or treated with olive oil or oleic acid) in response to negative (−60 mV) or positive voltages (+60 mV). Comparable values of current density were obtained from cells expressing the R272Q mutant channel after treatment (with either olive oil or oleic acid), demonstrating that this mutant was functionally rescued. Because the untreated mutant channel produced no current, the corresponding current density could not be calculated and displayed. For the R278W mutant channel, 2% olive oil treatment alone seems to improve protein trafficking (see Figure 2; Supplementary Figure 1). However, none of the treatments rescue the functionality of the R278W mutant channel (Figure 3). Again, because the mutant channel produced no current, the corresponding current density values could not be calculated. For the data shown in Figure 4, slight differences in current amplitude at positive and negative voltages were apparent, possibly because of the decreased open probability of CNG channels at negative voltages (Sesti et al., 1994). Although the WT channel currents are seemingly larger than those from the R272Q channel, no statistically significant differences were identified in any of the tested conditions, as determined by a one-way ANOVA (*p* > 0.05) (Supplementary Table 1). Together, the above data provide evidence that the R272Q the mutant protein retains ion-channel function and the mutation critically hampers its trafficking to the plasma membrane.

Numerous studies indicate that oleic acid contributes to increasing lipid droplet formation (Rohwedder et al., 2014; Nakajima et al., 2019; Eynaudi et al., 2021), and interactions between lipid droplets and endosomes are known to modulate membrane trafficking (Zehmer et al., 2009). In light of this, we hypothesize that an excess of free oleic acid might enhance the formation of vesicular lipids that enhanced trafficking of CNG channels. To probe or discard this hypothesis, we conducted experiments on non-transfected cells and compared the formation of intracellular lipidic structures in both treated and untreated cells. We selected Nile Red Lipid stain as the cellular lipid marker for the experiments, primarily due to its established efficiency in intracellular lipid staining (Collot et al., 2018). Additionally, its broad absorption and emission spectra enabled us to utilize both red and green light channels, thereby facilitating a more comprehensive analysis.

**FIGURE 5:**
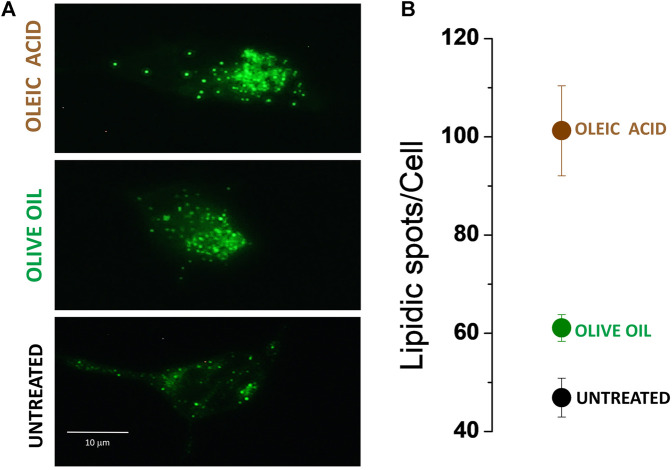
Nile red-stained HEK-293 cells. **(A)** Lipidic spots present under three different conditions: untreated cells; cells treated with olive oil; cells treated with oleic acid. **(B)** Mean ± SEM of lipidic fluorescence spot quantification. n = 5.

Micrographs in Figure 5A show that, indeed, un-transfected cells supplemented with olive oil and oleic acid experienced an increase in intracellular lipids. To quantitatively assess the number of lipidic spots expressed per cell, we generated binary images from 8-bit threshold adjusted images of individual cells and digitally counted the individual fluorescent spots (Size, pixel^2: 10-infinity). In Figure 5B, the mean and SEM of fluorescent spots detected in five individual cells are presented. We observed more fluorescent spots in cells treated with oleic acid (spot mean= 101.2, SEM = 9.17279) and olive oil (spot mean= 61, SEM = 2.701) than in control cells (spot mean= 46.8, SEM = 3.967). Fewer fluorescent spots were identified in olive oil-treated cells compared to oleic acid-treated cells, possibly due to either a lower concentration of oleic acid or a synergic effect with another compound in the oil. Further experiments are required to elucidate this relationship.

Although we cannot guarantee that all the lipidic spots detected by fluorescence are vesicular lipids potentially integrating organelle membrane-lipids, the results presented in this work support the fact that transport of membrane proteins like CNG channels is favored by olive oil and oleic acid supplementation.

Thus, the results presented in this work reveal that olive oil enhances membrane protein trafficking in cell culture.

## Discussion

In this work, we used cell cultures transfected with different CNG channel populations (wild-type or mutant) and individually supplemented them with olive oil or oleic acid. Then, we analyzed the effect on transmembrane protein location and function. According to our findings, channel functionality was be rescued when protein location was restored by supplementing the cell medium with olive oil or oleic acid (Figure 1). Our study aimed to investigate the effect of natural lipids, specifically olive oil and its main lipidic compound (the oleic acid), on two CNG channel mutants. Our intention was to explore the impact of these lipids, given that oleic acid has been shown to affect the functionality of other ion channels. To ensure the validity of our results, we included controls where we analyzed the effect of adding oleic acid or olive oil on the untransfected-cells. Our findings indicated that neither treatment affected cell morphology nor the function of the wild-type CNG channel.

While we acknowledge that olive oil is a complex mixture, our focus was on the potential of one of its components, oleic acid, known for its impact on ion channel functionality. Additionally, to our knowledge, similar experiments investigating the effects of natural lipids on CNG channels have not been previously reported, highlighting the novelty of our approach.

Hence, we believe that to promote protein trafficking, at least two critical factors must be considered. First, the topological localization of the mutated amino acid is crucial; unlike R272Q, R278W is situated near the intracellular side of the membrane, whereas R272W is located closer to the extracellular side (Li et al., 2017; Zheng et al., 2020; Zheng et al., 2022). Second, the nature of the amino acid side chain. Residue 272, arginine, is a basic amino acid with a positive charge, and it has been mutated to a polar residue (glutamine). In residue 278, the positive residue has been substituted by a non-polar amino acid (tryptophan). While preferential amino acid interactions with oleic acid have not been reported, it is evident that lipid interactions with proteins and amino acids are intricate and may depend on several factors, including cell membrane composition, the presence of other molecules, and the cellular and tissue context. Another crucial consideration is the extrinsic interaction with other proteins, lipids, and metabolites, as well as the interaction among amino acids (Aimon et al., 2014; Taberner et al., 2015; Hedger and Sansom, 2016; Liin et al., 2018; Duncan et al., 2020; Gu and de Groot, 2020).

Our results support the notion that a second arginine at S4 in CNG channels (R272) is not essential for maintaining channel functionality (Lopez-Rodriguez and Holmgren, 2012). However, R272 does modulate protein maturation, facilitating proper cellular localization. Conversely, residue 278 seems to be crucial for functionality, as none of the treatments were successful in rescuing it, even though our colocalization analysis indicated a slight effect on protein location (Figure 2).

Additional research is necessary to understand why our co-localization data does not exhibit a significant concentration dependence on olive oil or oleic acid. The concentrations of olive oil and oleic acid were chosen based on preliminary studies that identified an optimal range for effectiveness. The lack of apparent concentration dependence shown in Figures 1, 2 may be attributed to the use of a limited number of concentrations within this narrow range. However, we note that the representative current traces do demonstrate some dependence on additive concentration (Figure 3). In future studies, we hope to explore a wider set of concentrations to better understand their impact on protein trafficking and the resulting correlation coefficients. We also note that intracellular trafficking of the R278W mutant channel did appear to improve slightly in a concentration-dependent manner (Supplementary Figure 1, left panel). We will explore this further in future studies. We acknowledge that it will also be necessary to analyze if other components in olive oil might be additionally influencing trafficking of the mutant channels.

Channel interaction is not the only factor to consider when determining the channel location of membrane proteins; vesicular lipid transporters, which support intracellular protein trafficking, must also be examined. In this study we cannot definitely conclude whether the addition of olive oil or oleic acid specifically augmented lipid droplet biogenesis (Kuo et al., 2017) or increased intracellular vesicle trafficking. However, our results clearly demonstrate that olive oil or oleic acid addition caused more mutant channels to reach their destination in the plasma membrane (Figures 1, 2, 5).

Interestingly, and supporting our results, diets enriched with oleic acid increase levels of this fatty acid in plasma membranes (Bermudez et al., 2011; Ortega et al., 2012). In the cell, oleic acid can be integrated into the plasma membrane as a free fatty acid or it can bind to the thiol group of coenzyme A. This binding forms oleoil coenzyme A, which acts as an acyl synthase to form glycerophospholipids. Depending on the polar head, these glycerophospholipids can transform into phosphatidylcholine, phosphatidylserine, phosphatidylethanolamine, phosphatidylinositol, among other phospholipids (Antonny et al., 2015).

**FIGURE 6:**
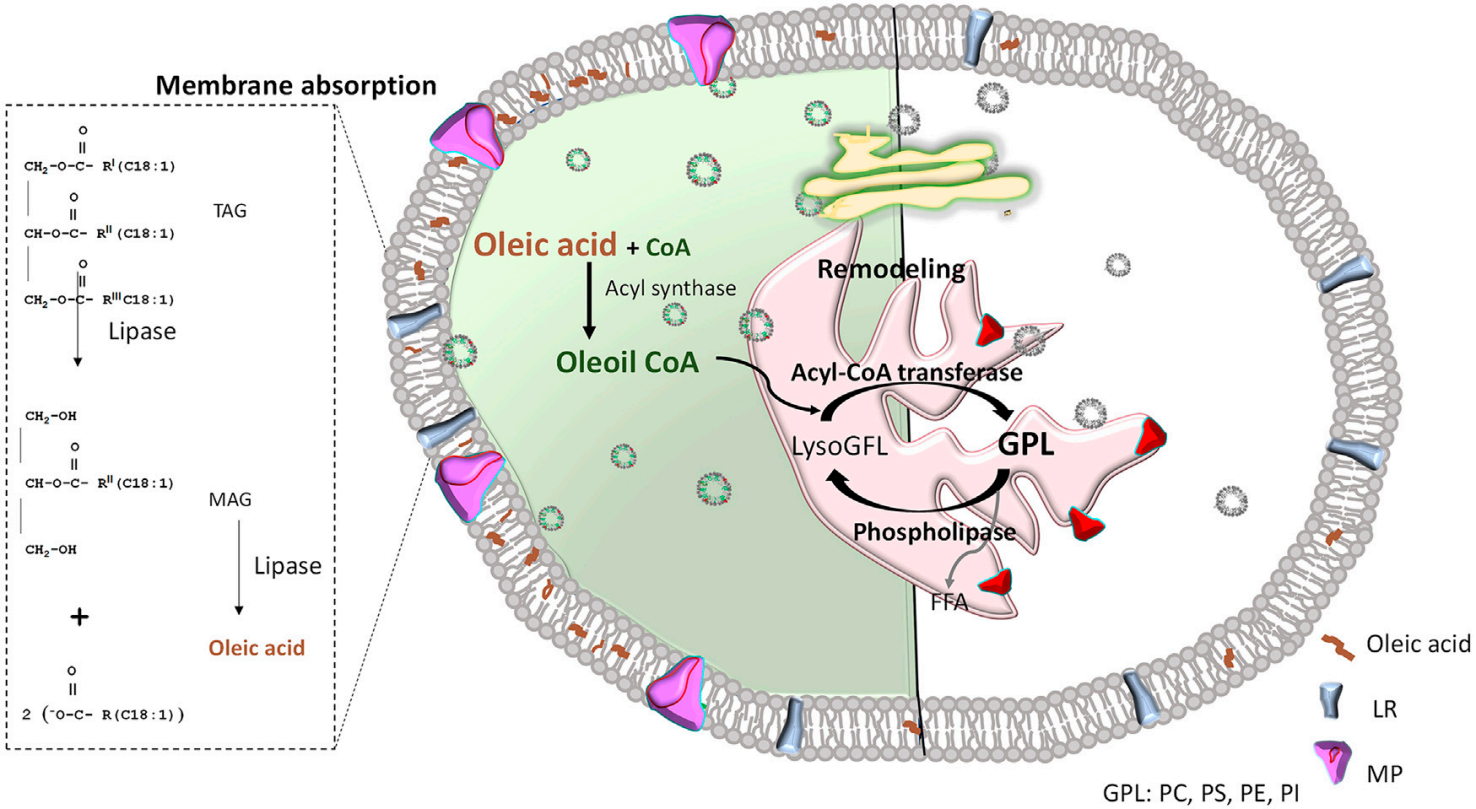
Effect of oleic acid inclusion in plasma membranes. Left: OA-treated; Right: untreated. Since oleic acid can enter the cell through lipid receptors (LR), it might be directly integrated into the plasma membrane as a free fatty acid. It can also be used as a phospholipid precursor that, in a reaction catalyzed by acyl-CoA transferase, becomes part of lyso-glycerophospholipid (LisoGPL) to form different glycerophospholipids (GPL), such as Phosphatidylcholine (PC), Phosphatidylserine (PS), Phosphatidylethanolamine (PE), Phosphatidylinositol (PI), among others. Then, phospholipids including oleic acid in their hydrophobic tails will integrate the endoplasmic reticulum membrane (shown in pink) and form protein transport vesicles, which can travel to the Golgi apparatus (shown in yellow) to ultimately fuse with a target membrane (i.e., plasma membrane), releasing the protein in a lipidic environment enriched with oleic acid (left side of represented cell). This allows greater curvature and facilitates the Efficient assembly of membrane proteins (MP). To simplify comparisons, a natural plasma membrane (not enriched with oleic acid) is illustrated on the right side. It is mainly formed by phospholipids with saturated fatty acids, inducing a more rigid membrane that hinders traffic and function of membrane proteins. FFA: free fatty acids; TAG: triacylglyceride; MAG: monoacylglycerols.

When oleic acid is incorporated into the phospholipids of the endoplasmic reticulum membrane, it likely becomes part of the protein transport vesicles, which are enriched with oleic acid (Alemany et al., 2010). These vesicles transport newly synthesized membrane proteins to the Golgi apparatus for posttranslational modifications, and they are capable of fusing with various target membranes, including the plasma, mitochondrial, and nuclear membranes. The “boomerang”-like shape of monounsaturated oleic acid alters membrane properties upon incorporation, as it enhances fluidity and prevents phospholipids from packing too tightly (Vicario et al., 1998; Perona et al., 2007; Lopez et al., 2014; Piccinin et al., 2019). This modification in lipidic composition could facilitate the assembly of hydrophobic and hydrophilic regions of transmembrane proteins, thereby promoting increased vesicular lipid formation and enhancing the trafficking of carried membrane proteins (Figure 6).

This research represents a significant contribution to our understanding of the effects of oleic acid and olive oil on protein trafficking. To the best of our knowledge, this is the first evidence demonstrating their role in promoting the trafficking of specific proteins. Interestingly, our findings align with previous studies that have indicated the ability of cells supplemented with fatty acids to utilize lipid components and synthesize triacylglycerol, resulting in their accumulation in lipid droplets. This phenomenon was previously evidenced using Raman spectroscopy and chemometrics (Paramitha et al., 2021).

Moreover, while prior research has provided indirect evidence suggesting the influence of lipid compounds on cellular processes involved in protein trafficking, our study offers direct evidence of this phenomenon. For example, oleic acid has been proposed as an inducer of autophagy, indirectly influencing protein trafficking by regulating protein degradation and transport (Jiang et al., 2017; Liu et al., 2023). Additionally, in astrocytic cells, oleic acid has been shown to be a potent inducer of lipid droplets, which are believed to form from microsomal membranes (Nakajima et al., 2019).

Our findings not only corroborate these previous observations but also provide novel insights into the mechanisms by which oleic acid and olive oil influence protein trafficking within cells.

## Conclusion

In summary, our findings highlight another possible beneficial aspect of consuming olive oil in our diet. Here we show that oleic acid functions as a modulator of membrane protein traffic and functionality. Giving the typical influence of lipidic environments on protein topological organization, we propose further exploration of saponifiable compounds in olive oil as potential lipochaperones for cell regulation. This avenue of research holds promise for gaining insights into diseases where protein trafficking to the plasma membrane is compromised. While our results suggest a potential role for olive oil in rescuing protein trafficking mutants, we recognize the need for caution in extrapolating these findings to advocate for olive oil consumption as a component of healthy diets. Our study represents a preliminary exploration into this area, and further research, including *in vivo* studies, is essential to fully understand any potential health benefits associated with olive oil consumption as a protein traffic inducer, as well as any possible negative consequences on channel biophysics.

## Data Availability

The original contributions presented in the study are included in the article/Supplementary Material, further inquiries can be directed to the corresponding authors.
