# Supplementary material for: Unlocking cellular traffic jams: olive oil-mediated rescue of CNG mutant channels
Source: Front Pharmacol. 2024 Jul 25;15:1408156. doi: 10.3389/fphar.2024.1408156 (PMC11306028; doi:10.3389/fphar.2024.1408156)
Supplement: Supplementary file 1 [file Table1.pdf]

Supplementary Table 1. One way ANOVA statistical analysis for CNG currents at -60 mV.

Sample	Sample Size	Mean	Standard Deviation	SE of Mean	
WT UNTREATED	4	69.73008	30.1211	15.06055	
WT OLIVE OIL 4%	4	105.20602	53.00185	26.50092	
WT OLIVE OIL 2%	4	85.42505	19.27997	9.63999	
WT OLIVE OIL 0.5%	4	92.17397	29.61169	14.80585	
WT OLEIC ACID 0.25%	4	48.51007	26.23964	13.11982	
WT OLEIC ACID 0.1%	4	85.05349	26.97539	13.4877	
WT OLEIC ACID 0.05%	4	108.01142	28.61891	14.30946	
R272Q OLIVE OIL 4%	4	61.44473	49.32405	24.66203	
R272Q OLIVE OIL 2%	4	92.058	44.77902	22.38951	
R272Q OLIVE OIL 0.5%	4	62.94536	21.4768	10.7384	
R272Q OLEIC ACID 0.25%	4	71.38532	21.65276	10.82638	
R272Q OLEIC ACID 0.1%	4	95.87287	11.83988	5.91994	
R272Q OLEIC ACID 0.05%	4	62.24329	36.73641	18.3682	
**Overall ANOVA**	**DF**	**Sum of Squares**	**Mean Square**	**F Value**	**Prob>F (P value)**
Model	12	16569.0398	1380.75332	1.27683	**0.2705**
Error	39	42174.4286	1081.3956		
Total	51	58743.4684			

Null Hypothesis: The means of all levels are equal.
Alternative Hypothesis: The means of one or more levels are different.
At the 0.05 level, the population means are not significantly different.
WT: wild type CNG channel. *R272Q*: mutant CNG-R272Q.
