# Supplementary figures and images for: Unlocking cellular traffic jams: olive oil-mediated rescue of CNG mutant channels
Source: Front Pharmacol. 2024 Jul 25;15:1408156. doi: 10.3389/fphar.2024.1408156 (PMC11306028; doi:10.3389/fphar.2024.1408156)

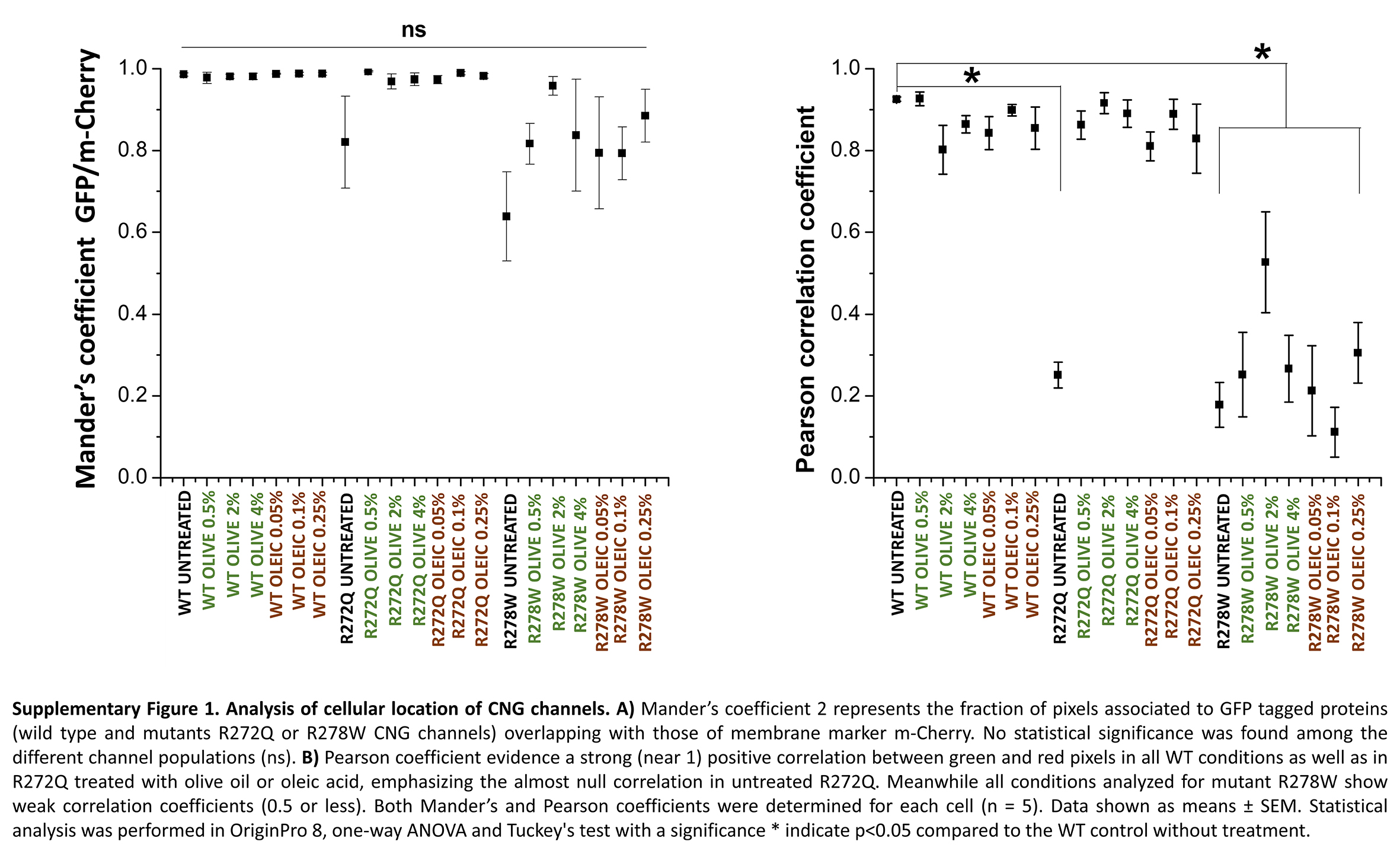

Supplement: Supplementary file 2 [file Image1.jpg]
